# Mitotically Active Plexiform Fibrohistiocytic Tumor

**DOI:** 10.1155/2013/547372

**Published:** 2013-03-27

**Authors:** Ebru Zemheri, Şeyma Özkanlı, Serkan Şenol, Filiz Ozen, Cigdem Ulukaya Durakbaşa, İlkin Zindancı, Hamit Okur

**Affiliations:** ^1^Department of Pathology, Istanbul Medeniyet University Goztepe Training and Research Hospital, Turkey; ^2^Department of Medıcal Genetics, Istanbul Medeniyet University Goztepe Training and Research Hospital, Turkey; ^3^Department of Pediatric Surgery, Istanbul Medeniyet University Goztepe Training and Research Hospital, Turkey; ^4^Department of Dermatology, Istanbul Medeniyet University Goztepe Training and Research Hospital, Turkey

## Abstract

Plexiform fibrohistiocytic tumor is an intermediate malignant tumor situated in superficial soft tissues. It affects children and young adults. The tumor is most commonly located on upper extremities, whereas involvement of back region is rare. Mitotic activity is generally low (~3/10 HPF). It is rare, but it can exhibit aggressive behavior, so total excision with clear surgical margins and long-term followup is necessary to detect local recurrence and metastases. We report a child with a solid mass on back region which was found to be a mitotically active plexiform fibrohistiocytic tumor (6/10 HPF) after excision. Plexiform fibrohistiocytic tumor (PFT) is a mesenchymal neoplasm of children, adolescents, and young adults. It is characterized by fibrohistiocytic cytomorphology and multinodular growth pattern. Clinically it is usually a slow-growing mass of upper extremities with frequent local recurrence and rare regional lymphatic and systemic metastasis (Fletcher et al. (2002), Enzinger and Zhang (1988), Remstein et al. (1999)).

## 1. Case Report

**Figure 1:**
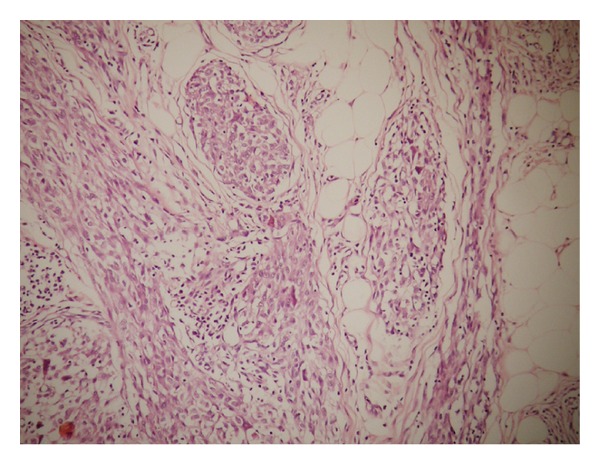
Plexiform fibrohistiocytic tumor showing multinodular mass in the subcutis (hematoxylin-eosin ×10).

**Figure 2:**
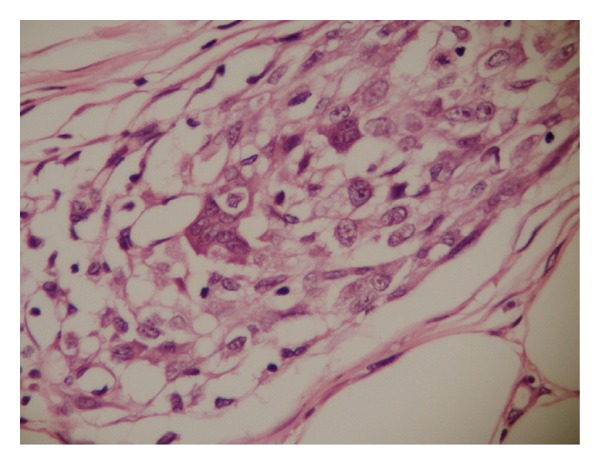
The tumor nodules with histiocyte-like and osteoclast-like giant cells and fibroblasts at the periphery (hematoxylin-eosin, ×40).

**Figure 3:**
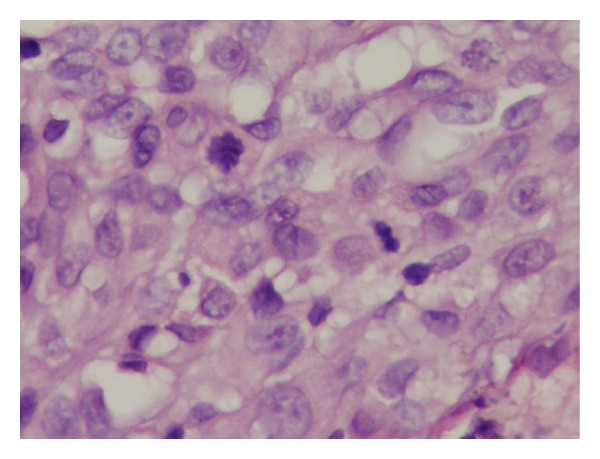
Two mitoses on HPF in some areas (hematoxylin-eosin, ×100).

**Figure 4:**
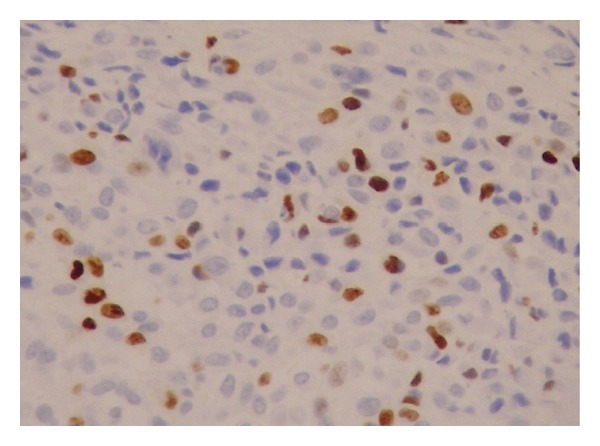
Ki 67 index was 15% (×20).

A 9-year-old male patient was admitted with the complaint of a slow-growing painless mass lesion in his left back region. On clinical examination, a firm, 1 cm nodule was detected and the lesion was excised totally. Macroscopically a solid mass measuring 1 cm in diameter was detected in deep dermis and subcutaneous tissue. There was no necrosis or hemorrhage. Microscopically, epidermis and upper dermis were normal. There were nodular clusters in deep dermal and subcutaneous areas and some of these were interconnected by fibroblast-like cells (Figure 1). The nodules were composed of mononuclear histiocyte-like cells and multinucleate giant cells. They were also wrapped by fibroblast-like cells (Figure 2). The mitotic count was 6/10 HPF. In some areas, 2/hpf mitosis was seen (Figure 3) with no atypical mitosis. Ki-67 index was 15% (Figure 4). There was no atypia, pleomorphism, or necrosis.

Immunohistochemically, there were strong positivity with CD68 in the histiocytic and giant cells and positivity with vimentin in the nodules and fibroblastic-like cells, positivity with SMA in some of which fibroblastic like cells were detected. The histopathological diagnosis was mitotically active PFT. The margins were not intact and a reexcision was recommended. A wider reexcision was performed with histologically proven complete excision. Followup of eleven months revealed no recurrence or metastasis.

## 2. Discussion

PFT is an uncommon tumor which was first described by Enzinger and Zhang [2]. The tumor is known to involve upper extremities usually the fingers, the hand, or the wrist whereas involvement of back region is rare [1–5]. Whereas PFT occurs most frequently in young females and the mean age at presentation is reported to be approximately 14.5 years [1, 4], two cases of congenital PFT were reported [6, 7].

The tumor size ranges from 0.3 to 8.5 cm [4]. These tumors most commonly present as solitary painless slow-growing lesions with a slightly raised overlying skin. Macroscopically, these tumors are multinodular masses are some of which poorly circumscribed, dermal, or subcutaneous masses [2, 4, 5].

Histologically, PFT is composed of multiple small nodules or elongated fascicles which are characteristically arranged in a plexiform pattern. Three different cell types are detected in variable amounts. These are spindle fibroblast-like cells, mononuclear histiocyte-like cells, and multinucleate giant cells [1, 2, 4, 5]. Different histologic patterns are reported. There is a fibrohistiocytic subtype which is composed of clusters of mononuclear histiocyte-like cells and multinucleated giant cells in a plexiform arrangement. The second one is a fibroblastic subtype which is composed mainly of elongated clusters and short fascicles of spindle fibroblast-like cells. The last is a mixed subtype which is composed of both fibrohistiocytic and fibroblastic patterns [4, 8]. Myxoid changes, cellular atypia, and pleomorphism are rarely seen. Mitotic activity is generally low (~3/10 hpf) and typical. In recurrent tumors, on the other hand, higher rates and atypical forms of mitoses are seen [1, 4, 5].

Immunohistochemically, the histiocytic cells stain for CD68, and the fibroblastic cells stain for vimentin and smooth muscle actin [4, 8, 9]. In addition negative staining for CD34, NK1/C3, factor XIIIa, and beta-catenin is useful to exclude of its mimics [10]. Leclerc-Mercier et al. showed a 46,X,del(X)(q13)[3]/46,XX[23] karyotype in cytogenetic analysis of one case and did not find any genomic anomaly in fluorescence in situ hybridization and array-comparative genomic hybridization analysis. They concluded that a molecular diagnosis of PFT was not reliable, because of lack of specific chromosomal hallmarks [11]. Ultrastructurally, PFT contains myofibroblast-like cells with abundant thin cytoplasmic filaments and histiocyte-like cells with lysosomes and multiple filopodia [4, 9]. Differential diagnosis of PFT include plexiform schwannoma, plexiform neurofibroma, fibrous hamartoma of infancy, deep benign fibrous histiocytoma, dermatofibroma, benign and malignant soft tissue giant cell tumor, and myofibromatosis [5, 8]. In addition, cellular neurothekeoma (CNT) and histiocytoid-predominant PFT share similar histopathologic and immunohistochemical features. Expression of MiTF may be a reliable marker for distinguishing CNT from PFT [12]. Plexiform fibrohistiocytic tumor is an intermediate-grade neoplasm. Incomplete excision may result in local recurrences. Enzinger and Zhang reported a local recurrence rate of 37.5%, while Remstein et al. reported an incidence of 12.5% [2, 3]. Regional lymph node and distant pulmonary metastases may occur [2, 3]. Solomao reported one case died of extensive metastasis of PFT [13]. In follow-up period, surveillance studies are performed to evaluate local recurrence and distant metastasis of malignant and intermediate tumors. There is no well defined interval and duration of follow up among various studies. Generally, vigorous surveillance continues for 3–5 years after treatment. It is not necessary to follow up with such surveillance in benign tumors [14]. In conclusion, PFT is an intermediate malignant tumor situated in superficial soft tissues. Because of the aforementioned malignant potential, the correct recognition is important for proper patient management and careful long-term followup.
